# EXPLORING CULTURALLY RESPONSIVE PHYSICAL ACTIVITY AS A RECRUITMENT AND RETENTION TOOL FOR AFRICAN AMERICAN WOMEN

**DOI:** 10.1093/geroni/igac059.2095

**Published:** 2022-12-20

**Authors:** Joyce Ogunrinde

**Affiliations:** University of Houston, Houston, Texas, United States

## Abstract

Physical activity (PA) is associated with lower cognitive decline and incident dementia for older adults. Yet, PA data (interventions) on older African American (AA) women, a population disproportionately affected by premature aging, are lacking. This limitation reduces the efficacy of PA to reduce cognitive decline, particularly for people of color and more so women of color whose race and gender create unique spaces for PA engagement. Although AA women desire to engage in PA, they face social, structural, and behavioral barriers to PA, challenges that parallel those faced in preventing premature aging (Li et al., 2018). Extant literature on AA women’s PA investigates social determinants of health (SDoH) and calls for more attention as to how these factors intertwine to shape these women’s PA over time (Fleury & Lee, 2006). Culturally responsive physical activity programs (CRPA) offer a framework for addressing these factors synergistically to promote PA in a way that is desirable to AA women. Specifically, CRPA provides a strength– based approach to explicate the ways PA can redress social, structural, and behavioral causes of cognitive decline and barriers to PA (cf., Joseph et al., 2020). The purpose of this poster is to explore the benefits of CRPA interventions on preventing cognitive decline. Implications include refining current models of PA as premature aging prevention measures by increasing our knowledge of the sociocultural factors shaping AA women’s aging and PA behavior and providing greater insight into the mechanisms for recruiting and retaining AA women into PA- based cognitive decline interventions.

